# Prevalence of rifampicin resistant *Mycobacterium tuberculosis* among presumptive tuberculosis patients in selected governmental hospitals in Addis Ababa, Ethiopia

**DOI:** 10.1186/s12879-019-3943-1

**Published:** 2019-04-04

**Authors:** Balew Arega, Fiqrte Menbere, Yitagesu Getachew

**Affiliations:** 1 Yekatit 12 Hospital Medical College, P.O.Box 257, Addis Ababa, Ethiopia; 2 Department of internal Medicine, Yekatit 12 Hospital Medical College, P.O. Box 257, Addis Ababa, Ethiopia; 3 Department of Public health research emergency management core process, Addis Ababa Administrative health office regional laboratory, P.O.Box 3-30378, Addis Ababa, Ethiopia

**Keywords:** MTB prevalence, Rifampicin resistance, MDR-TB, Addis Ababa, Ethiopia

## Abstract

**Background:**

Rapid detection of rifampicin resistance is essential for early management and prevention of transmission of multidrug-resistant tuberculosis (MDR-TB). We studied the prevalence of rifampicin resistance of *Mycobacterium tuberculosis* (MTB) among presumptive TB patients in Addis Ababa, Ethiopia.

**Methods:**

A retrospective cross sectional study was conducted in three referral hospitals and the regional laboratory in Addis Ababa city from March 2015 to October 2017. Data was collected by data-extraction sheet from registration books. It was analyzed using SPSS version 20. Statistically significant association was taken with *P* < 0.05.

**Results:**

A total of 12,414 (11,672 adults and 742 paediatrics) TB presumptive patients were included in the study. The overall prevalence of TB was 15.11% (1876/12414) in all age groups and 13.6%(101/742) among paediatric population. Rifampicin resistant TB was 9.9% (186/1876) in all TB confirmed cases and 7.9% (8/101) in paediatric TB patients. The prevalence of rifampicin resistant TB among new and previously treated was 7.6 and 27.4%, respectively. Sex (being female) and previous TB treatment were significantly associated with rifampicin resistant TB.

**Conclusions:**

Rifampicin-resistant TB is prevalent both among adult and paediatric TB patients. The strong association of rifampicin resistance with previous treatment in this study suggests the need to improve and monitor the treatment to limit the emergence of drug resistant TB.

## Background

The emergence and spread of multidrug-resistant tuberculosis (MDR-TB) is threat, which complicate diagnosis, treatment and control of the disease [1]. On average, 4.1% of newly diagnosed and 19% previously treated TB patients are estimated to MDR-TB worldwide in 2017 [2].

Empirical TB treatment without drug susceptibility testing (DST), which is a common practice in many developing countries, is believed to increase the day to day risk of transmission of drug resistant strains [3, 4]. Therefore, routine testing of all patients with TB is widely recognized as the most appropriate surveillance approach for monitoring trends in drug resistant TB [5].

WHO endorsed the Xpert MTB/RIF assay, which is a rapid and automated molecular system that detects both *M*.*tuberculosis* DNA and rifampicin-resistance (RR) associated mutations simultaneously. Research recognized that RR can be a surrogate marker for MDR-TB in more than 90% of the cases [6]. Hence, WHO recommends that RR-TB patients should be treated like patients with MDR-TB [7]. Initially, this technique was indicated for patients with TB/HIV co-infection, presumptive MDR-TB and paediatrics TB patients [8] but 3 years later of its implementation, it was recommended for all patients suspected of having TB [9]. Ethiopia ranks 10th among the high-TB-pandemic countries and 15th among the 27 high MDR-TB countries with more than 5000 estimated MDR-TB patients each year [10]. A recent national drug resistance survey conducted in Ethiopia reported a 2 and 11% prevalence of MDR-TB among new and pretreatment cases, respectively [11]. In addition, a meta-analysis and systematic review conducted in our country reported that drug resistant TB rates were stable in the last 10 years [12]. Drug resistant TB treatment is more complex than susceptible TB due to longer treatment time, increased toxicity and costs [13].

In Ethiopia, sputum smear microscopy is the commonly used laboratory diagnostics technique for TB. *Mycobacterium tuberculosis* culture, the gold standard test, is limited only to regional laboratories and primarily used for research purposes. The Xpert MTB/RIF assay is now going to be implemented in all health facilities, mainly in referral hospitals across the country since 2014, as per the recommendation of WHO [14]. This survey assessed the prevalence of RR-TB among presumptive TB patients diagnosed using Xpert MTB/RIF assay in selected governmental hospitals in the capital city of Ethiopia.

## Methods

### Study design, area and period

This was a health facility based retrospective cross-sectional study conducted from March 2015 to October 2017 in three referral governmental hospitals and the regional laboratory found in Addis Ababa Administrative health office. The study population were all TB presumptive (patients with clinical signs and symptoms suggestive of TB) patients who visited the study area during the study period.

### Laboratory investigation and data collection

A single sputum sample per patient for age greater than 6 years and a gastric aspirate sample in case of children less than this age group were used in all study facilities for the diagnosis of all presumptive TB patients using Xpert MTB/RIF assay. Samples were collected before the patients started anti-TB treatment. Samples were processed by GeneXpert MTB/ RIF assay. These were diluted and decontaminated, and the GeneXpert MTB/RIF assay was performed according to the manufacturer’s manual. Other laboratory diagnostic methods such as culture and acid fast bacilli (AFB) smear microscopy were not done for patients with *M*.*tuberclosis* negative result by Xpert MTB/RIF. Patients’ records that had incomplete data, e.g., age, gender, Xpert MTB/RIF results, HIV status, sample type, and TB treatment history were excluded from the study.

### Data processing, analysis

The data was entered in to EPI info version 7 and then exported to SPSS version 20 for analysis. Descriptive statistics was done for the calculation of frequencies of each variable. All the variables (Age, sex, previous TB treatment history, HIV) included in this study are traditional/known risk factors of drug resistance TB and included in the regression model irrespective of their value.

### Ethical issue

The study was approved by Yekatit 12 Hospital Medical College and Addis Ababa Administrative Health office ethical review committee (ERC). A letter of cooperation was written to each study areas authority and official permission was obtained. Informed consent was not sought from the study participants as it used secondary data. Confidentiality of the information collected was maintained by omitting their name and other personal identifiers from extraction sheet.

## Result

### Sociodemographic and clinical characteristics of the studied sample

Of the 15,099 presumptive TB patients that have submitted samples for TB diagnosis, 12,414 (82.2%) had complete data and were included in the study. Of these, 11,672 (94%) had age > 15 years and 742 (6%) had age ≤ 15 years (paediatric). The overall prevalence of MTB detected using Xpert MTB/RIF assay was 15.11% (1876/12414) in all age groups and 13.6% (101/742) among paediatric presumptive TB patients. The mean age of the tuberculosis diagnosed patients was 33.33 ± 13.99 years, of which 1107 (59%) were males and 769(41%) were females. Of 101 pediatric TB patients, 58/101 (57.4%) were in age range of 11–15 years. The HIV status was unknown for the majority of the patients (89.7%) and 11.7% (219/1876) had previously TB treatment history (Table 1).

**Table 1:** Sociodemographic and clinical characteristics of tuberculosis patients in Addis Ababa, Ethiopia (*N* = 1876)

Variables	Total TB patients	Percentage (%)
Sex		
Male	1107	59
Females	769	41
Age group		
0–5	30	1.6
6–10	13	0.7
11–15	58	3.1
16–30	863	46
31–45	584	31.1
46–60	250	13.3
> 61	78	4.2
History TB treatment		
Relapse	193	10.3
Failure	17	0.91
Lost to follow up	9	0.5
New cases	1657	88.3
HIV status		
Positive	117	6.7
Negative	77	4.1
Unknown	1682	89.7

*HIV* Human immunodeficiency virus

### Detection of rifampicin resistant M. tuberculosis

The overall prevalence of RR-MTB was 9.9% (186) in all TB diagnosed patients. In paediatric TB patients, 8 (7.9%) were RR-MTB positive. In this population (Table 2), previously TB treatment (*P* < 0.001) and female sex (*P* < 0.02) were found independently associated with RR-TB. In this population (Table 2), previously TB treatment (*P* < 0.001) and female sex (*P* < 0.02) were found independently associated with RR-TB. The prevalence of RR-MTB was almost equal among HIV positive and negative patients (14.5% vs 14.3% respectively). There were no significant differences in the prevalence of RR-MTB between patients under 15 years and above 15 years (*p* > 0.05).

**Table 2:** Prevalence of rifampicin resistant TB in TB patients based on sex, gender, age, treatment history, and HIV status in Addis Ababa, Ethiopia (*N* = 1876)

Variables	RR-TB N (%)	Not RR-TB N (%)	COR (95% CI)	AOR(95%CI)	*P* Value
Gender					
Male	95 (14.6)%	1012 (85.8)	reference		
Females	91 (11.8)	678 (88.2)	1.43 (1.06–1.52)	1.5 (1.10–1.99)	0.02
Age-group years					
0–15	8 (7.9)	93 (92.1)	reference		
16–30	94 (10.9)	769 (89.1)	1.421 (0.67–3.02)		
31–45	60 (10.3)	524(89.7%.)	1.33 (0.62–2.88)		
46–60	17 (6.8)	233 (93.2)	0.85 (0.35–2.03)		
> 61	7 (9)	71 (91)	1.15 (0.39–3.31)		
TB-treatment history					
Previously Rx cases	60 (27.4)	159 (72.6)	4.6(3.24–6.49)	4.7 (3.30–6.75)	0.001
New cases	126 (7.6)	1531 (92.4)	reference		
HIV status					
Positive	17 (14.5)	100 (85.5)	1.64 (0.956–2.81)		
Negative	11 (14.3)	66 (85.7)	1.61(0.832–3.12)		
Unknown	158 (9.4)	1524 (90.6)	reference		

*HIV* human immunodeficiency virus, *RR-TB* rifampicin resistant tuberculosis, *TB* Tuberculosis, *Rx* Treated, *COR* Crude Odd Ratio, *AOR* Adjusted Odd Ratio

## Discussion

In the present study, the prevalence of *M. tuberculosis* infection was found to be 15.11%. Other several studies reported quite a diverse magnitude of *M*. *tuberculosis* (15.4–25% [15–19] in the different parts of the country. This might be related to variation in risk for HIV acquisition, geographical variation, method of diagnosis, study setting or TB control practice.

The prevalence of *M*.*tuberclosis* among pediatrics TB Patients (13.6%) in the current study is comparable to a report from South Africa (13%) [20] and Uganda 14% [21]. Yet, it is lower than a study done in Southwest Ethiopia (31.7%) [22]. This substantial difference can be owing to the methods of diagnosis. While we used only Xpert MTB/RIF assay, the other study applied microscopy and culture and other molecular techniques for identification.

The existence of RR-TB is a serious health problem in the study population. The prevalence of RR-TB in this study is in line with previous studies in Ethiopia (9–10.3%) [17, 19] and Nigeria (12.1%) [23]. However, other studies in the country reported a lower (5.7–7.5%) [24, 25] and a higher (15.8–33.3%) [18, 26, 27] level of RR-TB. The variation of RR-TB across the country might be related to differences in patient selection, sample size (small sampling could generate higher resistance rate).

In the present study, the proportion of RR-MTB (7.9%) among paediatrics TB patients was consistent to two studies in China (6.9–8.3) [28, 29] yet higher than a study in Uganda (5.7%) [21] and in Thailand (5.1%) [30]. This study also found no significant differences (*P* > 0.05) for the presence of RR-TB among pediatrics (< 15) and adults. In the same way, other studies [31, 32] also support our findings. Since the largest source of drug resistance TB comes from adults, it seems reasonable to treat the paediatrics in similar regimen as of their contacts [33, 34]. However, children can acquire infection independently in the community [35], in case of TB high burden settings. Therefore, molecular typing of isolates from pediatrics TB patients and their potential contacts is essential to determine the source of transmission.

In the present study, RR-TB was significantly higher among previously treated patients compared to treatment naïve patients. This is in agreement with other studies in Ethiopia (18–19). However, the level of RR-TB among previously untreated cases (7.6%) in the analysis is higher than that reported in the latest Ethiopia national survey report (2%) [36], other study in the country (6.7%) [19], and in the WHO 2017 report (4.1%) [2]. The high prevalence of RR-TB among new TB cases in the current study suggests the existence of active person-to-person transmission or the existence of undiagnosed new RR-TB cases. In addition, drug resistance among previously untreated cases indicate the performance of TB control program in the past. The strict practice of direct observed therapy (DOTS) and DOTS-plus program currently run-through in Ethiopia is questionable.

According to our study, being female was identified as an independent risk factor for RR-TB. This is in agreement with the report from previous study in Ethiopia [27], another study in Georgia [37], Russia [38] and Estonian patients [39]. The prevalence of RR-TB among females might be related to socioeconomic factors, probably due to lack of control of financial resources at household levels), poor knowledge regarding TB as seen in other studies in country [40] and delay and poor health seeking behavior in females [41]. Since mothers are primary care giver of children in our country, it may increase risk of RR-TB among children. This is very valuable and instructive, and could aid in comprehension for health professionals and policy makers to address the problem. In this study we could not do microbiological confirmation of tuberculosis, phenotypic rifampicin resistance and resistance to other anti-TB drugs.

## Conclusion

In our setting, RR-MTB is prevalent both among adult and paediatric TB patients. Being female and previous treatment with anti-TB drugs was found significantly associated with rifampicin resistance. The strong association of rifampicin resistance with previous treatment suggests the need of an improved monitoring of treatment to limit the emergence of drug resistant MTB strains.

## Abbreviations

HIV: Human immunodeficiency virus
MDR-TB: Multidrug resistant tuberculosis
RR-MTB: Rifampicin resistant *Mycobacterium tuberculosis*
RR-TB: Rifampicin resistant tuberculosis
TB: Tuberculosis
WHO: World Health Organization
